# Association of physical activity with insulin resistance: A cross-sectional mediation analysis of the systemic immune-inflammation index in NHANES

**DOI:** 10.1097/MD.0000000000050465

**Published:** 2026-09-04

**Authors:** He Wang, Zhifeng Jia

**Affiliations:** a Department of Physical Education, Northeast Petroleum University, Daqing, Heilongjiang, China.

**Keywords:** insulin resistance, metabolic health, NHANES, physical activity, Triglyceride–glucose index

## Abstract

Higher levels of physical activity (PA) are associated with lower insulin resistance (IR), but the underlying mechanisms remain incompletely understood. Systemic inflammation may represent one pathway linking PA to IR. This cross-sectional study included 9088 adults aged ≥ 18 years from the 2005 to 2016 National Health and Nutrition Examination Survey. IR was assessed using the triglyceride–glucose index, total PA was calculated as metabolic equivalent minutes per week (MET-min/week), and the systemic immune-inflammation index (SII) was used as an inflammatory marker. Survey-weighted multivariable linear regression models were used to examine the association between PA and IR. Restricted cubic spline analyses were used to assess potential nonlinearity, and mediation analysis was performed to evaluate the mediating role of SII. Total PA was inversely associated with IR after multivariable adjustment (β [95% CI] = −0.089 [−0.127 to −0.052], *P* < .001). Restricted cubic spline analysis indicated a nonlinear association, with a clear inverse relationship between PA and IR at low-to-moderate PA levels and greater uncertainty beyond approximately 1922 metabolic equivalents of task-min/week. Mediation analysis showed that SII partially mediated the association between PA and IR (β [95% CI] = −0.005 [−0.009–−0.003], P < .01). Sensitivity analysis using multiple imputation for missing covariates showed a consistent inverse association between PA and IR after full adjustment. In a nationally representative US sample, higher PA levels were associated with lower IR, and systemic inflammation accounted for a small proportion of this relationship. The PA range associated with lower IR may help inform exercise-based strategies for the prevention and management of metabolic disorders.

## 1. Introduction

Insulin resistance (IR) is associated with an increased risk of type 2 diabetes, cardiovascular disease, metabolic syndrome, and obesity and is widely recognized as a common pathological basis for multiple metabolic disorders.^[1,2]^ Data from the Global Burden of Disease study indicate the global burden of metabolic syndrome and type 2 diabetes mellitus. Nonalcoholic fatty liver disease and cardiovascular diseases attributable to IR have increased markedly in recent years, imposing substantial pressure on individual health and healthcare systems.^[3]^ The triglyceride-glucose index (TyG), a convenient, reliable, and low-cost surrogate marker of IR, has been shown in multiple studies to outperform traditional IR assessment methods such as the homeostasis model assessment of IR and has therefore been widely used in epidemiological and clinical research.^[4]^

Physical activity (PA), as a nonpharmacologic intervention, has been widely shown to improve metabolic health. A large body of evidence suggests that regular moderate-to-vigorous PA can substantially reduce the risk of metabolic syndrome, type 2 diabetes mellitus, and cardiovascular disease by increasing energy expenditure, modulating lipid metabolism, and enhancing insulin sensitivity.^[5,6]^ However, the optimal PA dose for improving IR remains unclear, and the underlying mechanisms have not been fully elucidated.

In recent years, increasing evidence has highlighted the important role of inflammation in the development and progression of IR. Chronic low-grade systemic inflammation is considered a key mechanism that impairs insulin signaling pathways. Pro-inflammatory cytokines such as interleukin-6 (IL-6), tumor necrosis factor-α , and C-reactive protein can interfere with the phosphorylation of insulin receptor substrates, thereby attenuating insulin activity.^[7]^ PA may modulate inflammation by reshaping the immune microenvironment, suppressing the generation of pro-inflammatory immune cells, reducing the release of pro-inflammatory cytokines, and promoting the production of anti-inflammatory mediators.^[8,9]^ Nevertheless, evidence remains limited regarding whether PA improves IR through reductions in systemic inflammation.

Therefore, the present study aimed to examine the association between PA and IR in US adults using nationally representative National Health and Nutrition Examination Survey (NHANES) data. We further evaluated whether the systemic immune-inflammation index (SII), a composite biomarker reflecting systemic inflammatory and immune status, mediates this association. In addition, we explored the potential nonlinear dose-response relationship between PA and IR. We hypothesized that higher PA levels would be associated with lower IR and that SII would partially mediate this association.

## 2. Methods

### 2.1. Study population

**Figure 1.**
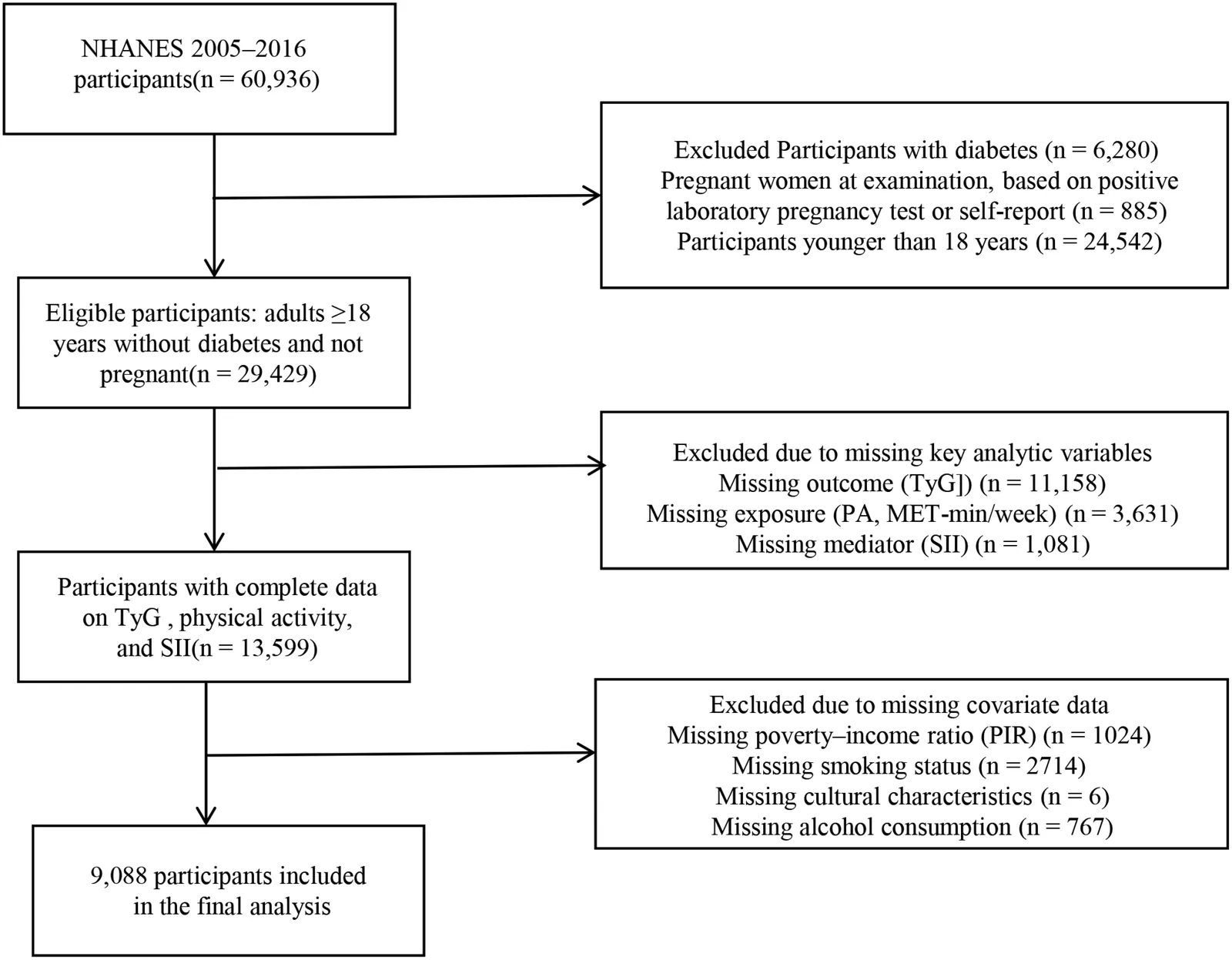
Flowchart of the inclusion of participants. MET = metabolic equivalent of task, NHANES = National Health and Nutrition Examination Survey, PA = physical activity, PLR = platelet-to-lymphocyte ratio, SII = systemic immune-inflammation index, TyG = triglyceride-glucose index.

NHANES is a nationally representative cross-sectional survey program conducted in the United States and administered by the National Center for Health Statistics (NCHS) of the Centers for Disease Control and Prevention (CDC). NHANES uses a stratified, multistage, clustered probability sampling design and collects a wide range of questionnaire, standardized physical examination, and laboratory data covering multiple health and nutrition indicators. All survey protocols are approved by the NCHS Research Ethics Review Board, and written informed consent is obtained from all participants. For the present analysis, we used data from NHANES cycles 2005 to 2016. We excluded individuals younger than 18 years, pregnant women, participants with diabetes, and participants with missing data on the TyG index, the SII, or PA-related metabolic equivalent of task (MET)variables. Participants with diabetes were excluded to reduce potential confounding related to disease-related behavioral modification and glucose-lowering treatment, both of which may influence PA and the TyG index. After these exclusions, 9088 participants were included in the final analytic sample. A detailed flowchart of participant inclusion and exclusion is presented in Figure 1.

### 2.2. Dependent variable

The dependent variable in this study was IR, assessed using the TyG index. The TyG index is a composite indicator derived from fasting triglyceride and fasting plasma glucose levels and has shown high accuracy for evaluating IR, with good applicability across diverse populations.^[10]^ The TyG index was calculated as:


TyG index=ln [TG(mg/dL)×FPG(mg/dL)/2]

### 2.3. Independent variable

The independent variable was PA, quantified as (MET). PA was assessed using the NHANES PA Questionnaire (PAQ), which is adapted from the World Health Organization (WHO) Global Physical Activity Questionnaire (GPAQ). The PAQ collects information on all types of PA performed during the past 30 days, including activity type, duration, intensity, and frequency. For each reported activity, a MET score corresponding to its type and intensity was assigned. The MET score was multiplied by the average duration and frequency over the past 30 days to obtain MET minutes over 30 days (MET-min/30 days) for each activity. MET-min/30 days values from all activities were then summed and divided by 4.29 (approximately 30/7) to estimate total weekly MET minutes (MET-min/week). Participants were further categorized into 2 PA levels according to whether they met the US Physical Activity Guidelines: low PA (<500 MET-min/week) and high PA (≥500 MET-min/week).^[11]^

### 2.4. Mediating variable

The mediating variable in this study was the SII. SII is a composite biomarker that reflects both systemic inflammatory activity and immune status and is calculated as:


$$\begin{array}{c} SII=\left(platelet\;count\times neutrophil\;count\right)/lymphocyte\;count \end{array}$$

SII provides an integrated measure of the balance between inflammatory and immune responses and has been suggested to have higher sensitivity and specificity than traditional inflammatory markers such as the neutrophil-to-lymphocyte ratio and platelet-to-lymphocyte ratio.^[12,13]^

### 2.5. Other covariate information

A set of covariates was included in the analysis, encompassing demographic, socioeconomic, and lifestyle characteristics. Potential covariates were selected a priori based on previous epidemiological studies and biological plausibility. Demographic characteristics included sex, age, and race/ethnicity. Socioeconomic factors included poverty-income ratio and educational level. Lifestyle factors included smoking status and alcohol consumption.

### 2.6. Statistical analysis

Because NHANES uses a complex multistage probability sampling design to obtain a nationally representative sample, all analyses incorporated primary sampling units, sampling weights, and stratification variables to derive nationally representative estimates. In accordance with NHANES analytic guidelines, survey-weighted analyses were conducted using the “survey” package in R. Because participants with missing data on the exposure, outcome, mediator, or covariates were excluded, a complete-case analysis was performed. Survey-weighted multivariable linear regression models were used to examine the association between PA and IR. The assumptions of linear regression were assessed, including linearity, normality, multicollinearity, and homoscedasticity. Restricted cubic spline (RCS) models were fitted within a linear regression framework to explore the potential nonlinear association between PA and IR, with 4 knots placed at the 5th, 35th, 65th, and 95th percentiles of PA. Mediation analysis was performed using the “mediation” package in R to evaluate the mediating role of SII in the association between PA and IR, with 95% confidence intervals estimated using nonparametric bootstrapping with 5000 resamples. No additional formal sensitivity analyses were prespecified beyond the multiple-imputation analysis described below.

## 3. Results

Table 1 shows that 9088 participants were included in the present study. As presented in Table 1, PA category was significantly associated with the TyG index and several sociodemographic factors, including sex, race/ethnicity, educational level, alcohol consumption, and SII (*P* < .05).

**Table 1:** Basic characteristics of survey respondents.

Variable	Total population (n = 9088)	Low physical activity (n = 1751)	High physical activity (n = 7337)	Statistic	*P*
Age, Mean (SE)	45.70 (0.31)	48.94 (0.50)	44.98 (0.33)	*t* = −7.70	<.001
Poverty-income ratio, Mean (SE)	3.13 (0.04)	3.09 (0.07)	3.14 (0.04)	*t* = 0.79	.434
TyG, Mean (SE)	8.57 (0.01)	8.64 (0.02)	8.55 (0.01)	*t* = -4.55	<.001
SII, Mean (SE)	523.78 (4.37)	563.99 (9.93)	514.85 (4.85)	*t* = -4.46	<.001
Sex, n (%)				*χ*^2^ = 100.62	<.001
1 (male)	4748 (51.86)	731 (40.70)	4017 (54.34)		
2 (female)	4340 (48.14)	1020 (59.30)	3320 (45.66)		
Race/ethnicity, n (%)				*χ*^2^ = 9.96	.025
1 (Mexican American)	1331 (7.46)	246 (6.79)	1085 (7.61)		
2 (Other Hispanic)	832 (4.88)	137 (3.74)	695 (5.13)		
3 (Non-Hispanic White)	4284 (71.66)	827 (72.55)	3457 (71.47)		
4 (Non-Hispanic Black)	1753 (9.52)	369 (10.70)	1384 (9.26)		
5 (Other race)	888 (6.47)	172 (6.22)	716 (6.53)		
Smoking status, n (%)				*χ*^2^ = 1.06	.448
0 (no smoking history)	5024 (55.05)	982 (56.19)	4042 (54.80)		
1 (smoking history)	4064 (44.95)	769 (43.81)	3295 (45.20)		
Educational level,n (%)				*χ*^2^ = 14.03	.009
1 (less than high school)	1879 (13.97)	366 (12.82)	1513 (14.22)		
2 (high school)	2043 (21.36)	427 (24.71)	1616 (20.62)		
3 (college or above)	5166 (64.67)	958 (62.46)	4208 (65.16)		
Alcohol consumption, n (%)				*χ*^2^ = 17.79	<.001
0 (no drinking history)	2345 (21.00)	546 (24.82)	1799 (20.15)		
1 (drinking history)	6743 (79.00)	1205 (75.18)	5538 (79.85)		

SII = systemic immune-inflammation index, TyG = triglyceride–glucose index.

Table 2 presents the results of the weighted multivariable linear regression analyses. A significant inverse association was observed between the TyG index and total PA expressed as metabolic equivalent minutes, and this relationship remained consistent after sequential adjustment for potential confounders. In the fully adjusted Model 3, which controlled for sex, race/ethnicity, smoking status, educational level, alcohol consumption, age, and poverty-income ratio, total PA remained significantly inversely associated with the TyG index (β [95% CI] = −0.089 [−0.127–−0.052]).

**Table 2:** Multivariable linear regression results for the association between physical activity and insulin resistance.

Variables	Model 1		Model 2		Model 3	
	β (95% CI)	*P*	β (95% CI)	*P*	β (95% CI)	*P*
Physical activity	−0.087 (−0.124–−0.049)	<.001	−0.092 (−0.129–−0.055)	<.001	−0.089 (−0.127–−0.052)	<.001

Model 1) Unadjusted model.

Model 2) Adjusted for sex, ethnicity, and age.

Model 3) Adjusted for sex, ethnicity, smoking status, educational level, alcohol consumption, age, and poverty-income ratio.

**Figure 2.**
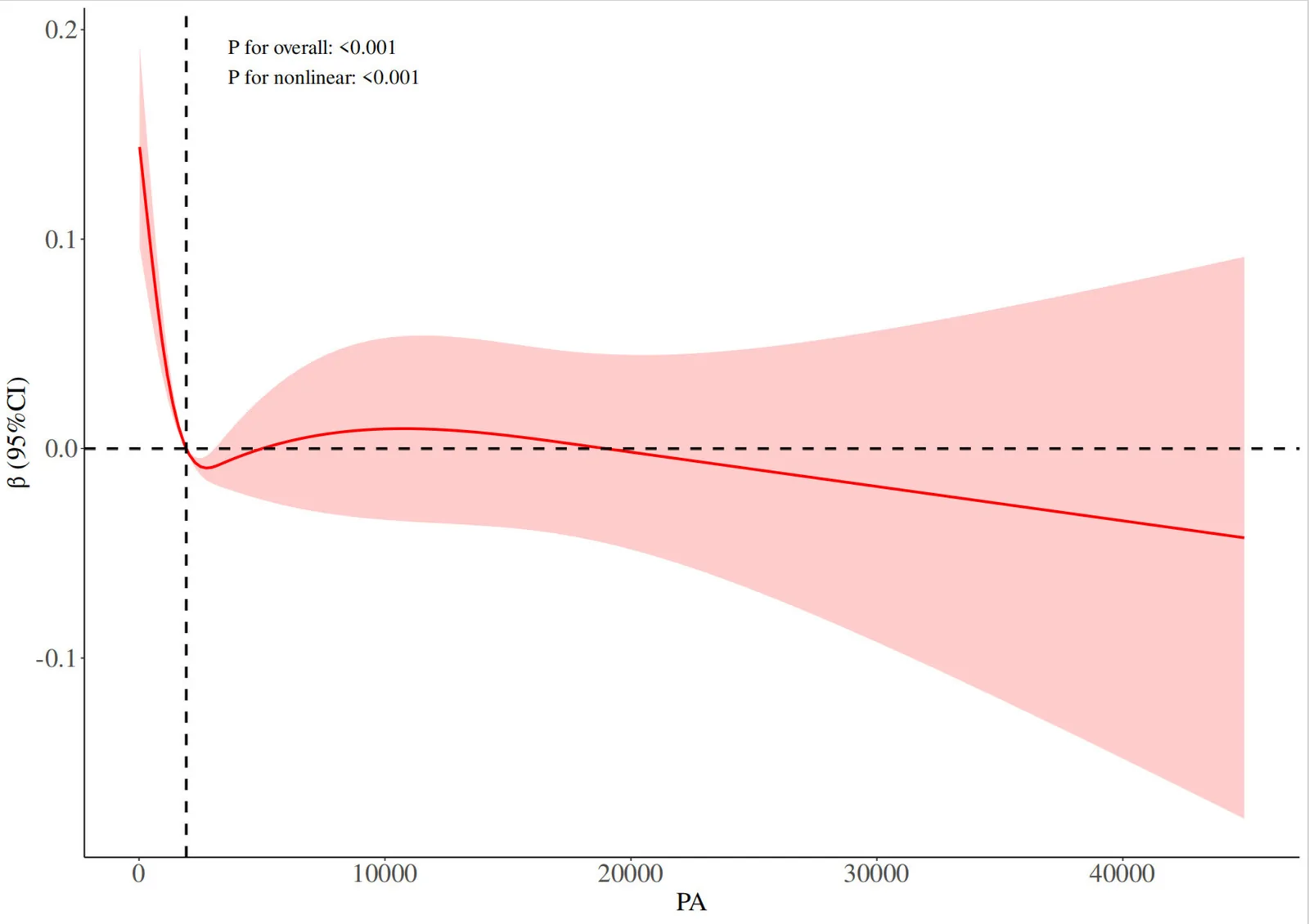
Restricted cubic spline curve for the association between total physical activity (MET-min/week) and the TyG index. MET = metabolic equivalent of task, TyG = triglyceride-glucose index.

In addition, restricted cubic spline analysis (Fig. 2) revealed a nonlinear association between total PA (MET-min/week) and the TyG index. At low-to-moderate activity levels (0–1922 MET-min/week), PA was inversely associated with the TyG index; beyond approximately 1922 MET-min/week, this inverse association became less evident.

**Figure 3.**
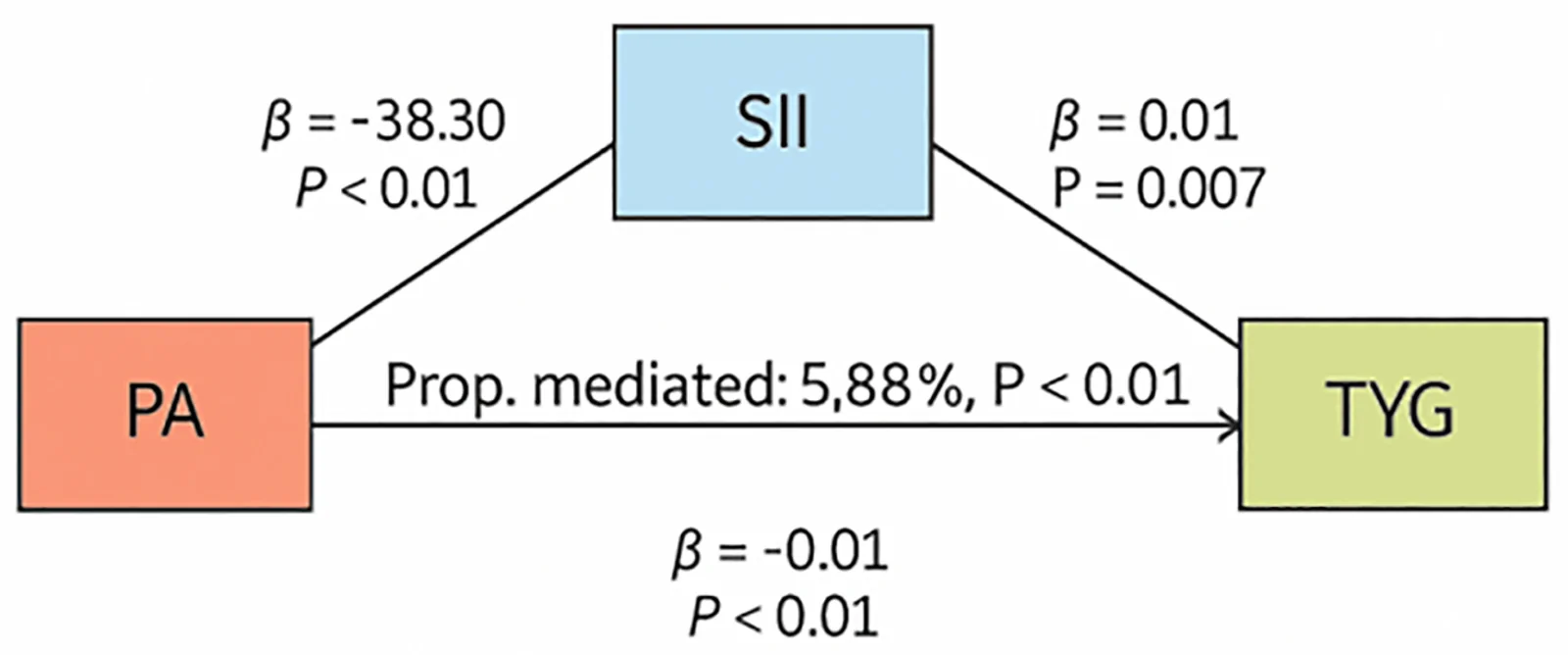
Mediation model for the association between physical activity and the triglyceride–glucose index through the Systemic Immune-Inflammation Index. PA = physical activity, SII = systemic immune-inflammation index, TyG = triglyceride-glucose index.

Mediation analysis showed that SII significantly mediated the association between PA and the TyG index, with a mediation effect estimate of β (95% CI) = −0.005 (−0.009–−0.003), accounting for approximately 5.8% of the overall association (Fig. 3).

Sensitivity analysis supported the robustness of the conclusions. After excluding participants with missing data on PA, the TyG index, and SII, we performed multiple imputation for missing values in all covariates. After adjustment for confounding factors in Model 3, PA remained significantly and negatively associated with the TyG index (*P* < .001), as shown in Table 3.

**Table 3:** Association of physical activity with insulin resistance after multiple imputation.

Variables	Model 1		Model 2		Model 3	
	β (95% CI)	*P*	β (95% CI)	*P*	β (95% CI)	*P*
Physical activity	−0.108 (−0.142 ~ −0.075)	<.001	−0.088 (−0.122 ~ −0.054)	<.001	−0.085 (−0.119 ~ −0.051)	<.001

Model 1: Unadjusted model.

Model 2: Adjusted for sex, ethnicity, and age.

Model 3: Adjusted for sex, ethnicity, smoking status, educational level, alcohol consumption, age, and poverty-income ratio.

## 4. Discussion

Using large-scale, population-based data from NHANES, this study examined the association between PA and IR and explored the potential mediating role of inflammation in this relationship. Higher PA levels, expressed as metabolic equivalent minutes, were significantly and inversely associated with the TyG index, and this association remained robust after successive adjustment for multiple covariates. Restricted cubic spline analyses further indicated a nonlinear pattern, whereby the inverse association between PA and the TyG index became uncertain beyond approximately 1922 MET-min/week. In addition, mediation analysis suggested that inflammatory status partially mediated the association between PA and the TyG index. Taken together, these findings provide evidence for an inverse association between PA and IR and suggest that systemic inflammation may represent one possible pathway involved in this relationship.

### 4.1. Association between PA and IR

Insulin resistance (IR) is a fundamental pathophysiological factor underlying metabolic disorders such as obesity, hypertension, and type 2 diabetes.^[14,15]^ PA, as an active lifestyle intervention, is widely recognized for its beneficial effects on metabolic health. Previous studies have reported that individuals with higher PA-related MET levels have a significantly lower risk of developing IR and that middle-aged and older adults who regularly engage in PA exhibit more favorable IR profiles than their inactive counterparts.^[16]^ Combined aerobic and resistance training has also been shown to improve IR in older adults.^[17]^ Consistent with these findings, the present study observed a significant inverse association between PA and IR, as assessed by the TyG index. This association may be explained by several biological mechanisms through which PA modulates IR, including enhanced skeletal muscle mass and function, improved glucose metabolism, and reductions in inflammation and oxidative stress.^[18,19]^ During exercise, skeletal muscle glucose uptake is enhanced through insulin-independent pathways, largely through increased translocation of glucose transporter type 4 to the cell membrane, a process that remains effective even in individuals with IR.^[20]^ At the same time, aerobic and resistance training improve mitochondrial content and function, augment fatty acid oxidation, and reduce the accumulation of lipid intermediates, thereby alleviating lipotoxicity and oxidative stress and helping restore insulin signaling.^[21]^ PA also reduces visceral adiposity and favorably alters adipokine profiles, for example, by increasing adiponectin levels and reducing leptin resistance, which promotes fatty acid oxidation and glucose utilization in peripheral tissues.^[22]^ In addition, exercise activates the endocrine function of skeletal muscle and stimulates the release of myokines such as IL-6 and irisin, which promote adipose tissue browning, increase energy expenditure, and help maintain metabolic homeostasis in the liver and skeletal muscle, collectively contributing to improved insulin sensitivity.^[23]^

However, the metabolic benefits of PA do not appear to increase indefinitely and may vary according to dose. In this study, restricted cubic spline analysis indicated a nonlinear association between PA (MET-min/week) and the TyG index, with attenuation of the inverse association when PA levels exceeded approximately 1922 MET-min/week. This observed value is slightly higher than the PA level corresponding to the World Health Organization recommendation of 150 to 300 minutes of moderate-intensity aerobic activity per week for general health benefits.^[24]^ Acute high-intensity or excessive exercise may induce a transient energy deficit and stress response, including increases in pro-inflammatory cytokines (e.g., IL-6 and TNF-α) and cortisol levels, which may temporarily attenuate insulin signaling and blunt further improvements in IR.^[25]^ In addition, inflammatory responses to very high PA volumes may follow a U-shaped pattern: moderate PA suppresses inflammation, whereas excessive PA may trigger immune stress responses and elevate inflammatory markers, thereby partially offsetting the beneficial effects of exercise on IR. However, this observed value should be interpreted as a potential inflection point in the current dataset rather than as a strict PA threshold, particularly given the cross-sectional design.

### 4.2. Mediating role of the SII

Inflammatory responses play a pivotal role in the development of IR, particularly under conditions of physical inactivity, which promote chronic low-grade systemic inflammation. Pro-inflammatory cytokines such as TNF-α, IL-6, and C-reactive protein are persistently elevated in this context and can activate key signaling pathways, including c-Jun N-terminal kinase (JNK), thereby impairing the normal phosphorylation of insulin receptor substrates.^[6]^ This, in turn, attenuates downstream phosphoinositide 3-kinase/protein kinase B (Akt) signaling, directly disrupting insulin signal transduction and reducing insulin sensitivity. Inflammation can also aggravate hepatic IR by inducing hepatic lipid accumulation and promoting endoplasmic reticulum stress.^[26]^ In the present study, SII was used as a quantitative indicator of inflammatory burden. SII is derived from platelet, neutrophil, and lymphocyte counts and therefore reflects both the balance between pro-inflammatory and anti-inflammatory components and the overall systemic immune-inflammatory status. Compared with single inflammatory markers, SII may provide a more integrated reflection of systemic immune-inflammatory status and may therefore be useful for characterizing the relationship between PA and IR.

Our results showed that inflammation, as indexed by SII, partially mediated the association between PA and IR, with the indirect effect accounting for 5.8% of the total effect. Although the mediation proportion was modest, this finding suggests that systemic inflammation may represent one potential pathway linking PA and IR. From a physiological perspective, regular moderate-to-vigorous PA can reduce the secretion of pro-inflammatory cytokines from monocytes and macrophages, thereby attenuating the inhibitory effects of TNF-α, IL-6, and related mediators on insulin signaling pathways and facilitating glucose uptake and utilization in skeletal muscle and liver.^[27]^ Moreover, exercise-induced skeletal muscle contraction can stimulate the release of myokines with anti-inflammatory properties, suppress sustained activation of the nuclear factor kappa B signaling pathway, and ameliorate the systemic low-grade inflammatory milieu.^[28]^ These mechanisms may partly explain why higher PA was associated with lower IR in the present study. In addition, PA may improve adipose tissue function by reducing visceral fat accumulation and lowering the secretion of pro-inflammatory adipokines, which may further alleviate inflammation-related impairment in insulin sensitivity.

## 5. Limitations and future directions

Several limitations should be acknowledged. First, because this study was based on cross-sectional NHANES data, temporal ordering and causality cannot be established, and reverse causality cannot be excluded. Second, PA was assessed using the self-reported Physical Activity Questionnaire (PAQ), which may be subject to recall bias and misclassification, potentially resulting in exposure measurement error. Third, although we adjusted for multiple covariates, residual confounding from unmeasured or imprecisely measured factors cannot be ruled out. In addition, participants excluded because of missing data may have differed systematically from those included in the final analysis, and these differences could not be fully assessed using the available data. Although multiple imputation was performed for missing covariates, potential selection bias and reduced representativeness could not be eliminated. Fourth, the mediation analysis should be interpreted cautiously because the mediator and outcome were assessed within a cross-sectional framework, preventing confirmation of the temporal sequence required for stronger mediation inference. Finally, we did not conduct subgroup analyses to examine whether the association between PA and IR differed across important subgroups, such as age, sex, adiposity, or metabolic status. Future studies should explore subgroup-specific patterns and verify these findings in prospective cohorts to provide more precise evidence for tailoring PA recommendations aimed at improving metabolic health.

## 6. Conclusion

In conclusion, higher PA was associated with lower IR in US adults, and this association appeared nonlinear. SII partially mediated the association between PA and IR. These findings may improve understanding of the potential role of inflammation in this relationship and warrant further investigation in longitudinal studies.

## Author contributions

**Conceptualization:** He Wang.

**Data curation:** He Wang.

**Funding acquisition:** Zhifeng Jia.

**Project administration:** Zhifeng Jia.

**Resources:** Zhifeng Jia.

**Writing – review & editing:** Zhifeng Jia.

**Writing – original draft:** He Wang.
